# The Use of Panitumumab-IRDye800CW in a Novel Murine Model for Conjunctival Squamous Cell Carcinoma

**DOI:** 10.1167/tvst.11.7.23

**Published:** 2022-07-27

**Authors:** Gun Min Youn, Ayden G. Case, Trent Jarin, BaoXiang Li, Aditi Swarup, Andrea Naranjo, Charbel Bou-Khalil, Jacqueline Yao, Quan Zhou, Marisa E. Hom, Eben L. Rosenthal, Albert Y. Wu

**Affiliations:** 1 Stanford University School of Medicine, Stanford, CA, USA; 2 Department of Ophthalmology, Stanford University School of Medicine, Stanford, CA, USA; 3 Trinity College of Arts and Sciences, Duke University, Durham, NC, USA; 4 Department of Otolaryngology–Head and Neck Surgery, Stanford Hospital and Clinics, Stanford, CA, USA; 5 Department of Neurosurgery, Stanford University School of Medicine, Stanford, CA, USA; 6 Department of Otolaryngology–Head and Neck Surgery, Vanderbilt University Medical Center, Nashville, TN, USA

**Keywords:** conjunctiva, squamous cell carcinoma, panitumumab-IRDye800, fluorescent probes

## Abstract

**Purpose:**

Conjunctival squamous cell carcinoma (SCC) is a sight-threatening ocular surface malignancy with the primary treatment modality being surgical resection. To evaluate surgical imaging modalities to improve surgical resection, we established a novel murine model for conjunctival SCC to demonstrate the utility of panitumumab-IRDye800, a fluorescently labeled anti-epidermal growth factor receptor (EGFR) antibody.

**Methods:**

NOD-scid IL2Rgamma^null^ (NSG) mice received subconjunctival injection of UM-SCC-1 or SCC-9, head and neck SCC cell lines. On tumor growth, mice were injected with Panitumumab-IRDye800CW, and imaged with a small animal imaging system and optical coherence tomography (OCT). Immunohistochemistry for SCC markers were used to confirm tumor origin.

**Results:**

Seventy-five percent (N = 4) of the UM-SCC-1 group developed aggressive, rapidly growing tumors that were P40 and EGFR positive within two weeks of inoculation. The SCC-9 tumors failed to demonstrate any growth (N = 4). Ocular tumors demonstrated high fluorescence levels with a tumor to background ratio of 3.8.

**Conclusions:**

Subconjunctival injections are an appropriate technique to create in vivo models for assessing treatment modalities and novel therapies in conjunctival SCC.

**Translational Relevance:**

This model demonstrates Panitumumab-IRDye800CW's utility in the ophthalmic setting and suggests that clinical trials may be warranted.

## Introduction

Conjunctival squamous cell carcinoma (SCC) is the most common ocular surface malignancy and is associated with sunlight exposure and human papilloma virus.^1^^,^^2^ Although the current standard of care is a surgical excision of the tumor, recurrence rates can be significant, with some studies noting rates as high as 30%.^3^^–^^5^ If the tumor cannot be controlled, conjunctival SCC can spread to the anterior chamber, orbit, and brain and potentially metastasize via hematologic or lymphatic routes.^6^ To avoid these situations, enucleation may be required, which poses a significant burden to patients and makes conjunctival SCC a potentially sight-threatening condition.^7^

Although mouse models serve a critical role in the research setting, no such models exist for conjunctival squamous cell carcinoma.^8^ Hwang et al.^8^ did establish a rabbit model for this disease through a SCC subconjunctival injection, demonstrating that subconjunctival injections can be used to create animal models for ocular surface tumors. Since then, several mouse conjunctival melanoma models have been successfully created by combining this technique with various ocular and cutaneous carcinoma cell lines.^9^^–^^11^

In other fields such as otolaryngology, mouse models have been used to test the efficacy of near-infrared fluorescent imaging agents such as Panitumumab-IRDye800CW, a fully humanized monoclonal IgG2 antibody.^12^ This probe binds to the epidermal growth factor receptor (EGFR), which conjunctival SCC is known to express, and has a 6.3 mm maximum penetration depth and a 774/789 nm excitation/emission maximum.^2^^,^^13^^–^^16^ In this study, we aimed to establish a novel mouse model for conjunctival SCC via a subconjunctival injection and use this model to determine the feasibility of Panitumumab-IRDye800CW in ophthalmology.

## Methods

### Cell Culture

Two commercially available head and neck squamous cell carcinoma lines UM-SCC-1 and SCC-9 were used because of the lack of established, readily available conjunctival SCC lines. All lines were routinely screened for mycoplasma and mouse pathogens and cleared for murine experiments. UM-SCC-1 was kept in Dulbecco's modified Eagle medium (Gibco, Grand Island, NY, USA), 10% fetal bovine serum (Omega Scientific, Tarzana, CA, USA), 1% non-essential amino acids (Gibco), and 1% penicillin/streptomycin (Gibco). SCC-9 was kept in DME F/12 media (Gibco), fetal bovine serum (Omega Scientific), 400 ng/mL hydrocortisone (Sigma-Aldrich, St. Louis, MO, USA) and 1% penicillin/streptomycin (Gibco). On the injection day, cells were collected with trypsin (Gibco), washed with phosphate-buffered saline solution (PBS) (Gibco), counted, and suspended in PBS. Suspended cells were placed on ice and resuspended before each injection.

### Animals and Injection

Eight NOD-scid IL2Rgamma^null^ (NSG) mice, aged 15 to 25 weeks, were obtained from Stanford University's mice colonies and housed under Institutional Animal Care and Use Committee guidelines. This study was approved by the Stanford University Institutional Animal Care and Use Committee and complies with the ARVO Statement of the Use of Animals in Ophthalmic and Visual Research.

**Figure 1.**
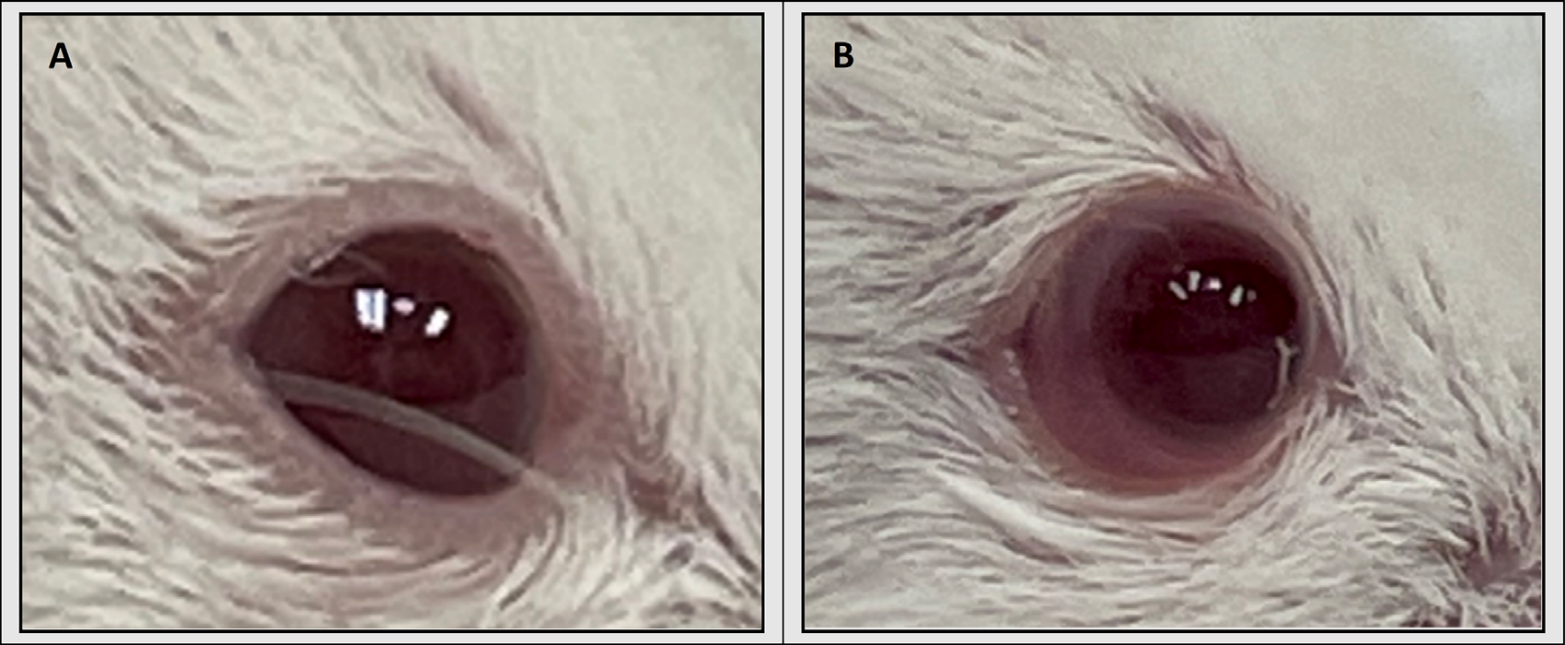
(**A**) Image of right eye of NSG mice before injection. (**B**) Image of right eye immediately after injection with 5 × 10^5^ UM-SCC-1 cells suspended in 5 µL of PBS.

Mice were evenly divided into two groups and received 5 µL injections of either UM-SCC-1 or SCC-9 cells (5 × 10^5^) into the right subconjunctival space using a 32-gauge Hamilton syringe (Hamilton Company, Reno, NV, USA) (Fig. 1). The left eye was used as a control and received 5 µL subconjunctival injection of PBS. Subconjunctival injection was verified by lack of tumor cell reflux along with the presence of a temporary conjunctival edema. Prior to injection, mice were anesthetized with either an intraperitoneal injection of combination 80 mg/kg body weight ketamine and 8 mg/kg body weight xylazine (AKORN, Lake Forest, IL, USA) or isoflurane (VetOne, Boise, Idaho, USA) (induce 3%-4%; maintain 1%-2%).

Mice were monitored for clinical signs of illness and symptoms of severe ocular pathology including corneal ulceration/perforation, conjunctivitis/blepharitis, or infection. Mice demonstrating proptosis due to tumor growth were imaged and euthanized. All mice were euthanized within four weeks of injection regardless of tumor growth status. Exenteration was performed and tissue was collected for pathological analysis. Necropsy was conducted and the liver, lung, spleen, and kidneys removed and analyzed for metastases.

### Fluorescent Imaging With Panitumumab-IRDye 800

Once the experimental endpoint was achieved, mice were given 20 µL of panitumumab-IRDye800CW through intraperitoneal injection. Panitumumab-IRDye800CW is produced by incubating panitumumab (Amgen, Thousand Oaks, CA, USA; 147 kDa) with 1.1 kDa IRDye800CW-NHS ester (LI-COR Biosciences, Lincoln, NE, USA) for two hours at 20°C in the dark.^17^ Mice were imaged immediately, 24, 48, and 72 hours post-injection using the PEARL Triology (LI-COR Biosciences, Lincoln, NE, USA). A tumor to background ratio (TBR) was calculated by measuring the mean fluorescent intensity from the area of interest and normal adjacent tissue using the PEARL's integrated instrument software (ImageStudio; LI-COR Biosciences).

### OCT Imaging

Before euthanasia, mice were anesthetized as per the above regimen, and artificial tears were applied to both eyes. Tumor growth was measured using optical coherence tomography (OCT) (HRA2-Spectralis; Heidelberg Engineering, Heidelberg, Germany).

### Histology and Immunostaining

Mice were euthanized and exenteration was performed. Tissue for frozen section was fixed in 4% paraformaldehyde overnight at 4°C and placed in increasing percentages of sucrose solution (from 10–30%). Frozen tissue blocks were created by embedding the tissues in OCT Compound Media (Sakura Finetek, Torrance, CA, USA) and sectioned into 10–12 µm slices with a LEICA CM3050S (Leica Biosystems, Deer Park, IL, USA) for immunohistochemistry. Standard formalin-fixed paraffin embedded (FFPE) tissue blocks were also created. Slides were imaged for panitumumab-IRDye800CW using the Odyssey imaging platform (LI-COR Biosciences).

Hematoxylin and eosin slides with 10-12 µm frozen sections were fixed in 10% formalin for 15 minutes and washed in water, stained with Hematoxylin for 3 minutes to stain nuclei and washed with water. Slides were then immersed in blueing agent for 40 seconds, washed with water again and immersed in 95% ethanol for 30 seconds. Cytoplasm was stained with EOSIN for 2-3 minutes and quickly dipped in water. Slides were immersed in 95% alcohol and absolute alcohol 3 times, 10 dips each and mounted with Cytoseal 60 (Thermo Fisher Scientific, Waltham, MA, USA) using coverslips. Standard Hematoxylin and eosin slides with 4 µm FFPE sections were also created.

Frozen sections were placed onto a hot plate at low temperature for 5 minutes and then allowed to dry for another 5 minutes. After drying, 4% paraformaldehyde was applied to the sections for 5 minutes followed by PBS wash three times. Blocking buffer solution containing PBS and 1% goat serum (Thermo Fisher) were added to the mounted sections and incubated at room temperature for 1 hour. After blocking, the following primary antibodies were used and then incubated with blocking buffer solution at 4°C overnight: EGFR (monoclonal rabbit anti-mouse; Thermo Fisher), Anti-p40 (polyclonal rabbit anti-mouse; Sigma-Aldrich). After washes in PBS, sections were incubated in Alexa Fluor 488 goat anti-Rabbit IgG polyclonal antibodies (Thermo Fisher) for 2 hours at room temperature followed by incubation in DAPI for 5 minutes to stain for nuclei. Slides were then washed three times with PBS and mounted with Cytoseal 60 (Thermo Fisher) on coverslips. Imaging was performed using Leica Thunder Imager EM Cryo CLEM Microscope (Leica Biosystems) at 2.5X, 10X and 20X magnification.

### Statistical Analysis

Wilcoxon signed-rank tests were used to compare panitumumab-IRDye800CW tumor to background ratios. One way analysis of variance was utilized to compare TBRs between panitumumab-IRDye800CW imaging days. All statistical analysis was performed with RStudio Version 1.4.1106.

## Results

### Tumor Growth

**Figure 2.**
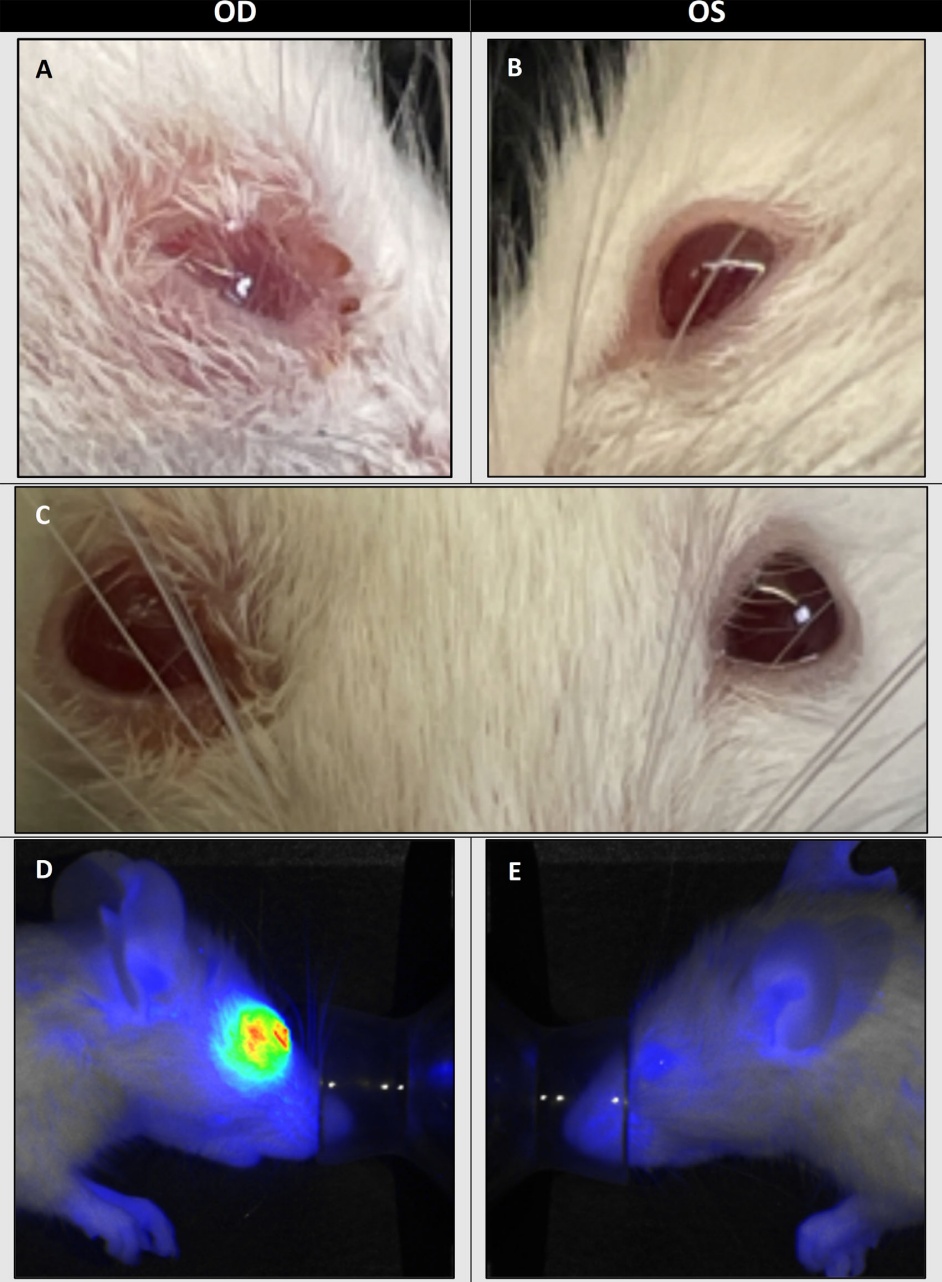
(**A**) Image of right eye with tumor growth. NSG mice were injected with 5 × 10^5^ UM-SCC-1 cells into the right eye, with the earliest tumors detected 10 days after injection. (**B**) Image of left (control) eye injected with 5 µL of PBS. (**C**) Frontal image of tumor growth in the right eye with no changes to the left eye. (**D**) Panitumumab-IRDye800CW uptake in treated right eye with red color representing higher intensity. (**E**) Panitumumab-IRDye800CW uptake in control left eye.

**Figure 3.**
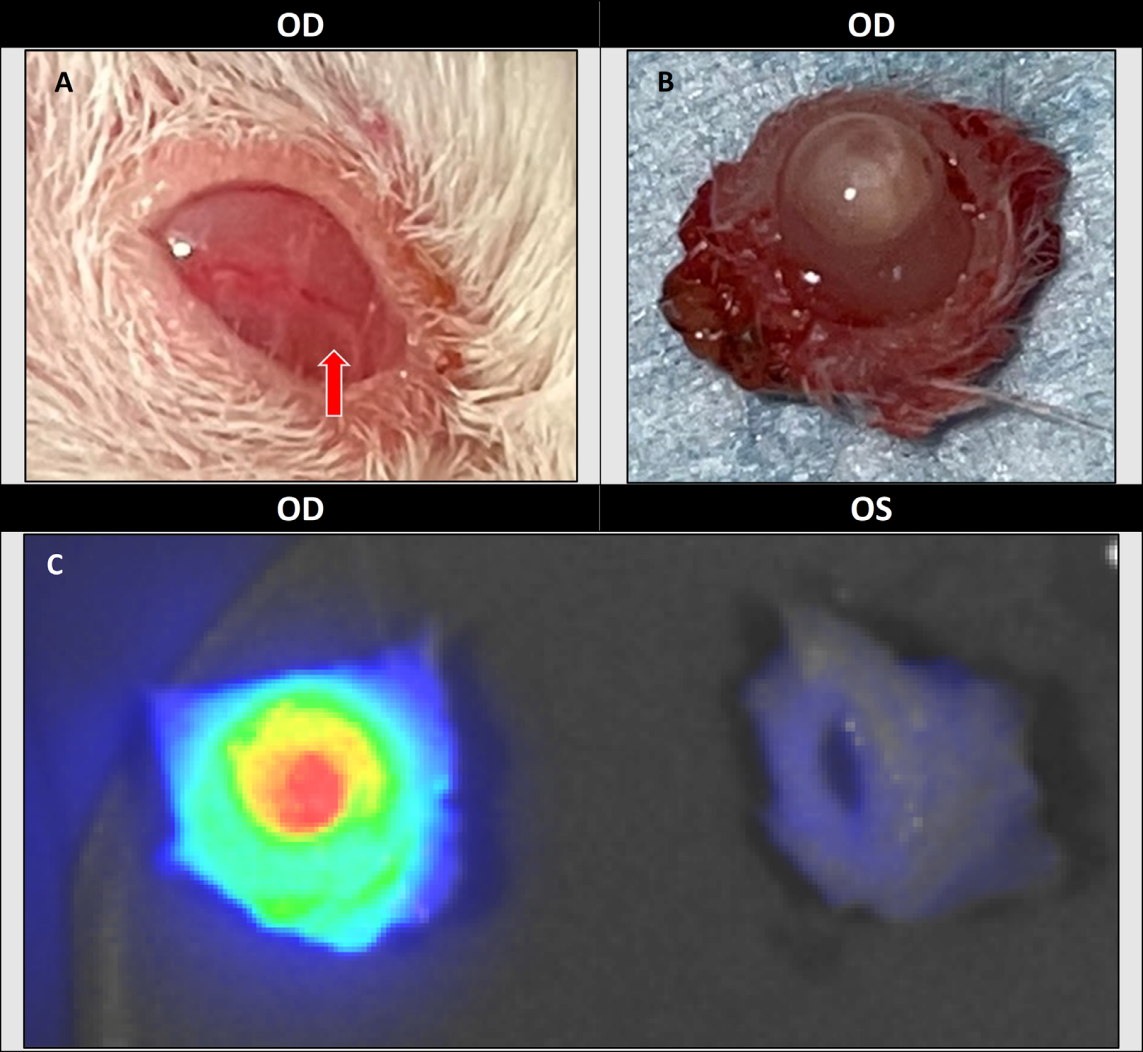
(**A**) Image of right eye with lower eyelid retracted to expose tumor growth. (**B**) Exenteration of the right eye with tumor growth. NSG mice were injected with 5 × 10^5^ UM-SCC-1 cells into the right eye, and mice were euthanized on average 18 (SD 3.5) days after injection. (**C**) Panitumumab-IRDye800CW uptake in right eye injected with UM-SCC-1 versus control left eye injected with 5 µL of PBS. In Panitumumab-IRDye800CW staining, *red* represents higher intensity.

To develop a murine model for human conjunctival squamous cell carcinoma, we subconjunctivally injected mice with the UM-SCC-1 line, (N = 4). 75% of these animals developed sizeable tumors within three weeks, with the earliest ocular growth detected 10 days post- injection (Figs. 2 and 3). Consistent with finding of other investigators developing conjunctival tumor models tumor measurements were unfeasible.^11^ Mice in the UM-SCC-1 group were euthanized on average 18 (SD 3.5) days post injection.^11^ No growth was observed in the SCC-9 group and all mice were euthanized 28 days post-injection. Necropsy found no metastases in the liver, lung, spleen, and kidneys.

### Panitumumab-IRDye800CW Imaging

Our second aim was to identify if panitumumab-IRDye800CW could be utilized in an ophthalmological setting. All eyes with tumors in the UM-SCC-1 group demonstrated strong fluorescent signal from the panitumumab-IRDye800CW with a mean tumor-to-background ratio of 3.81 (SD 1.40), which was significantly higher than control eyes with a TBR of 1.32 (SD 0.29) (p = 0.0039), (Fig. 5). There was no significant difference in TBRs between days in either the UM-SCC-1 group (p = 0.214) or the SCC-9 group (p = 0.381).

### Optical Coherence Tomography

**Figure 4.**
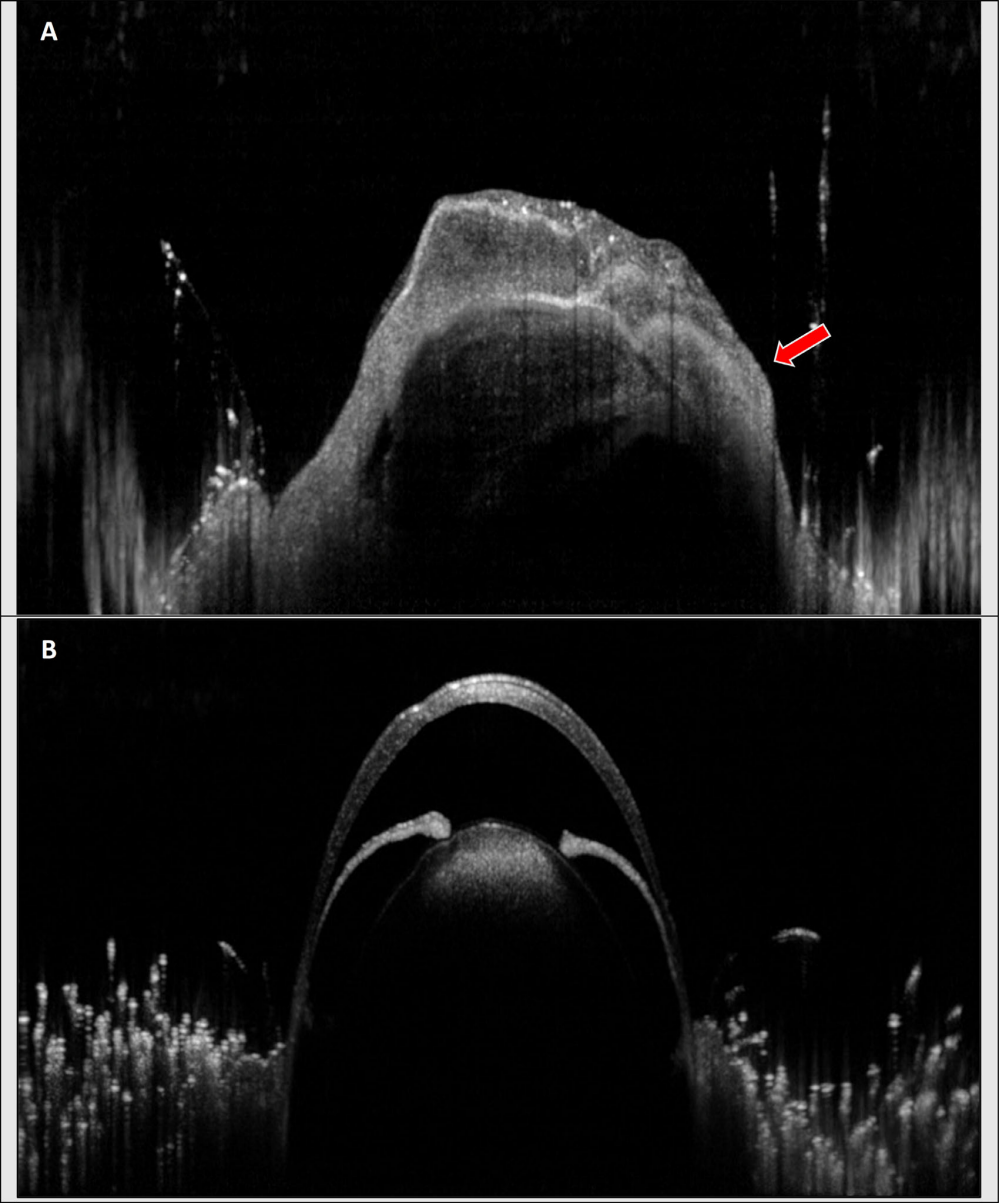
(**A**) OCT image of right eye treated with 5 × 10^5^ UM-SCC-1 cells. OCT was performed immediately prior to euthanasia, which occurred on average 18 (SD 3.5) days after injection. (**B**) OCT imaging of left (control) eye treated with 5 µL of PBS.

OCT imaging identified tumor growth in the nasal region in 66% (n = 2) of the UM-SCC-1 injected, tumor bearing eyes (Fig. 4). OCT imaging showed no difference between SCC-9 injected and control eyes.

### Slides Results

H&E staining of the tumor bearing UM-SCC-1 eyes showed pleomorphic nests of cells with high levels of eosinophilic cytoplasm that invaded the epithelial basement membrane (Table 1). Imaging the panitumumab-IRDye800CW demonstrated that the tumor was fluorescent with strong signal in both the subconjunctiva, as well as the anterior and posterior chamber, confirming that the subconjunctival injection led to rapid, invasive growth. Immunohistochemistry was performed to verify the tumor's origin. P40 and EGFR staining was positive, in all tumor-bearing eyes, confirming that the tumor was SCC (Table 2).

**Table 1.** Representative magnified hematoxylin-eosin-stained sections that demonstrate nests of pleomorphic cells with high levels of eosinophilic cytoplasm

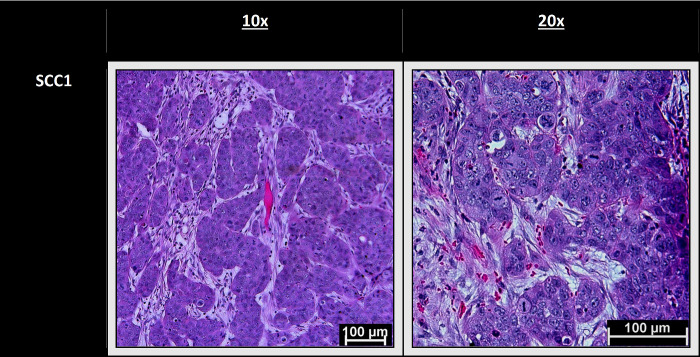

**Table 2.** Representative Images of H&E, p40, EGFR, and Panitumumab-IRDye800CW Staining of UM-SCC-1 Sections. In p40 and EGFR Staining, *green* Represents Higher Intensity, Whereas *blue* Indicates DAPI Staining. In Pan800 Staining, *red* Represents Higher Intensity

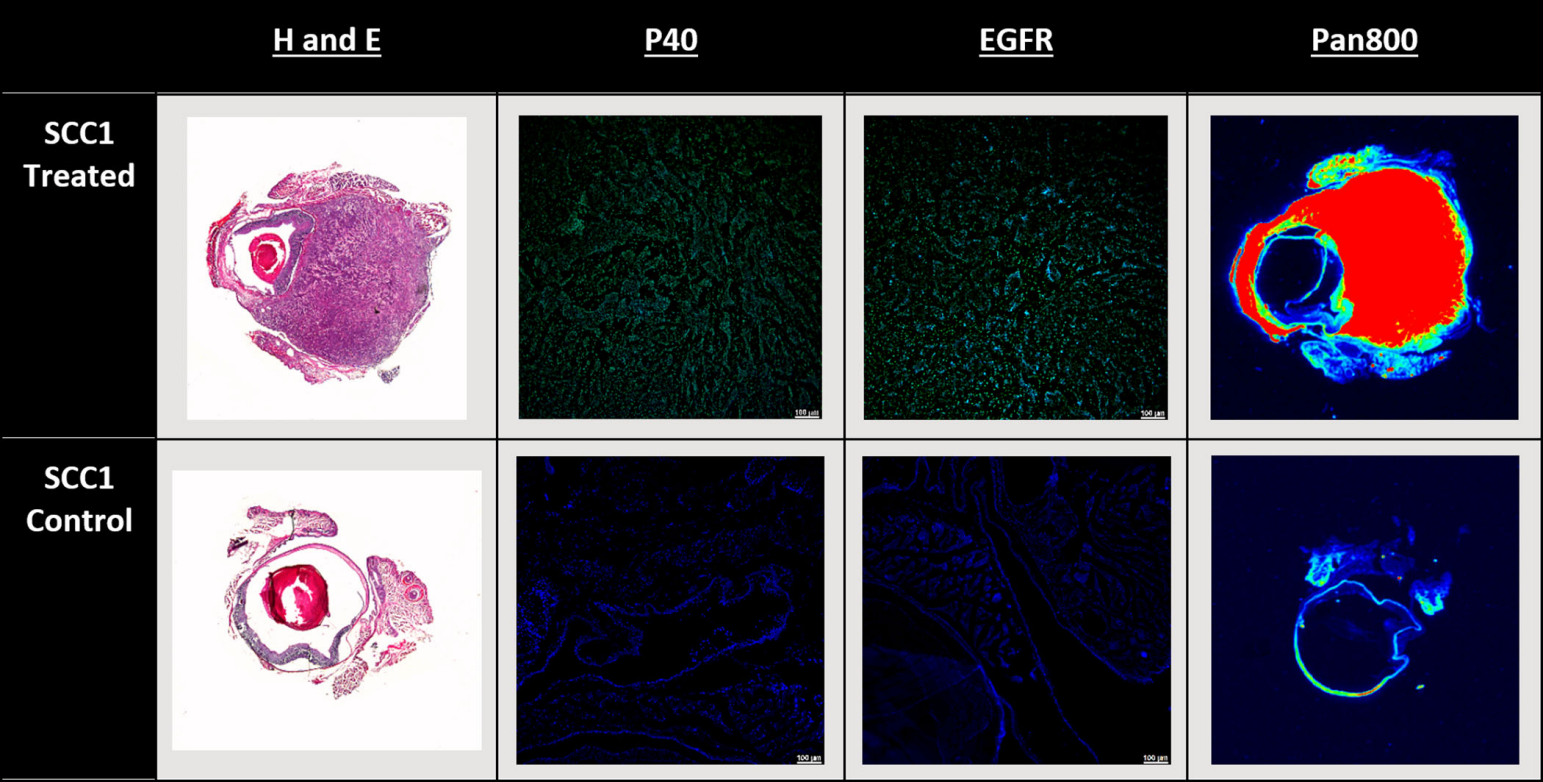

## Discussion

Fluorescent-guided surgery (FGS) has become a major area of innovation over the past decade with several approved agents and more than a dozen agents in clinical trials.^18^^,^^19^ To evaluate the role of FGS in ocular malignancies, we developed a mouse model for conjunctival SCC and then demonstrated the potential of panitumumab-IRDye800CWs for in vivo imaging. The development of conjunctival SCC is marked by an initial conjunctival intraepithelial neoplasia that is contained in the epithelium.^20^ If left untreated, epithelial basement membrane invasion occurs, which is a hallmark of invasive SCC that has the potential to invade the anterior chamber and orbital tissue.^20^^,^^21^ Thus one major advantage of this subconjunctival technique is the creation of a model that more closely mimics invasive SCC, which has a higher recurrence rate and leads to vision loss.^7^ Our model is consistent with other investigators, who used the same technique to successfully create murine conjunctival melanoma models.^10^^,^^11^

One major advantage of our model is its rapid tumor growth, because the majority of mice injected with the UM-SCC-1 cell line developed tumors within two weeks. This is consistent with other subconjunctival models that found growth within five days to two weeks.^8^^–^^11^ However, despite positive results in the UM-SCC-1 line, the SCC-9 line failed to show any growth, underscoring the importance of cell line selection. These different outcomes may potentially be explained by significantly lower levels of EGFR expression in SCC-9 relative to UM-SCC-1. In 2018, Khaznadar et al.^22^ found that compared to other HNSCC lines, SCC-9 had relatively lower rates of EGFR expression and phosphorylation—the latter of which is critical in the mediation of the signal cascade and cell growth. Additionally, another study found that compared to SCC1, SCC9 had an almost threefold lower rates of EGFR expression.^23^ Because EGFR plays a functional role in tumorigenesis and is implicated in both head and neck, as well as conjunctival SCC, lower levels of EGFR may have contributed to failed xenograft uptake and growth. Additionally, we used epithelial tumor lines instead of primary conjunctival SCC lines, which may have played a role in the failure of SCC-9. However, as noted by Schlereth et al.,^10^ both conjunctival and epidermal cells are similar in terms of development, embryonic origin, receptors, and lymphatic drainage. When combined with the dearth of readily accessible conjunctival SCC lines, SCC lines with similar properties are among the best available options to create an orthotopic model for conjunctival SCC. Histologically, our model demonstrated a tumor with nests of pleomorphic cells, high levels of eosinophilic cytoplasm, and positive staining for both p40 (an established tumor marker) and EGFR.^24^

Immunohistochemistry (IHC) also found positive EGFR staining in the UM-SCC-1 line. EGFR is a receptor tyrosine kinase that when activated increases cell differentiation, proliferation, and survival and has been used to target numerous breast, otolaryngological, and colorectal malignancies.^12^^,^^25^^–^^29^ In 2012, Hope et al.^28^ and Heath et al.^12^ used panitumumab-IRDye800CW, a humanized, fluorescently labeled antibody that binds EGFR to image microscopic head and neck cancer in an orthotopic murine mode and was able to detect high fluorescence levels at the malignant site.

**Figure 5.**
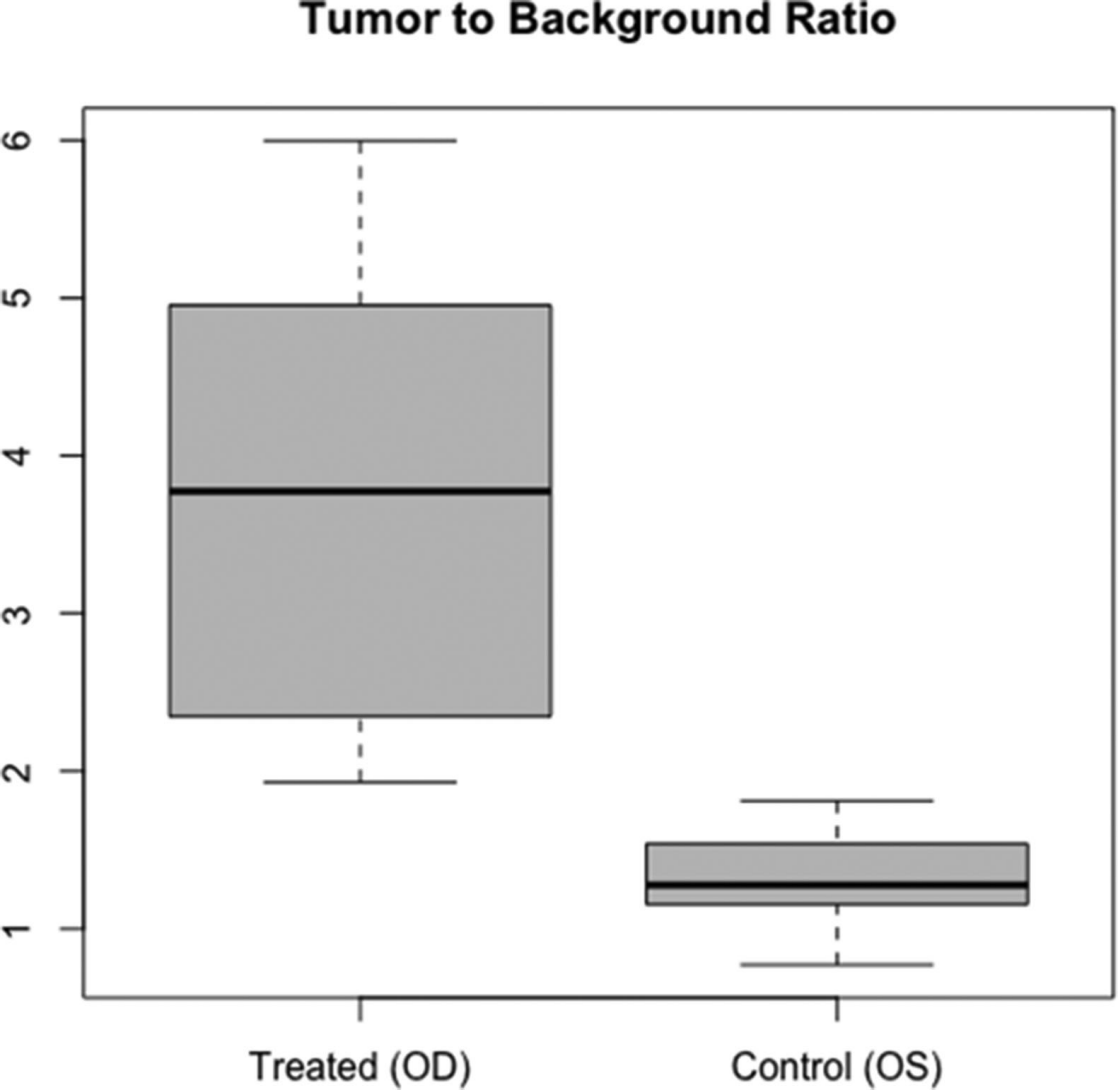
Panitumumab-IRDye800CW Tumor to Background Ratios for NSG Mice Treated With UM-SCC-1 Cells in the Right Eye and PBS in the Left Eye. Panitumumab-IRDye800CW Demonstrates Significantly Greater Fluorescence in Treated Eyes (*P* = 0.0039).

Since then, this intraoperative fluorescent system has evolved and is being used in clinical trials across the country for head and neck, pancreatic, and brain cancer.^13^^,^^17^^,^^30^^–^^34^ Our experiment clearly demonstrates that in in vivo models, systemic panitumumab-IRDye800CW injection is able to adequately penetrate ocular tissue and provide enough fluorescent signal to demarcate pathologic areas (Fig. 5). This is underscored by the high TBR we found within mice that demonstrated tumor growth (mean 3.81), which is crucial in limiting false positives and optimizing image-guided surgery.^28^

From a clinical perspective, even though current intraoperative systems used to image Panitumumab-IRDye800CW are not optimized for ocular tissue, Panitumumab-IRDye800CW closely mimics indocyanine green's emission and excitation properties, which is used for various ophthalmic procedures.^12^^,^^35^ Given indocyanine green's widespread use in ocular imaging, it's possible that eventually, systems that already exist can use Panitumumab-IRDye800 in image-guided surgery and improve outcomes.

### Future Directions

Although our in vivo model provides a proof of concept for the potential application of FGS in ophthalmology, further work is needed before clinical applications. Future experiments could be based on the creation of a human conjunctival SCC line followed by subconjunctival injection and analysis of FGS application. Additional cell lines could also be potentially used to identify cell line characteristics that lead to graft uptake success and failure.

### Limitations

First, our proof-of-concept study had a small sample size, yet the robust tumor growth in the majority of the UM-SCC-1 line combined with positive immunohistochemistry and statistically significant difference in TBRs indicate that our technique creates a viable model for conjunctival SCC. Second, we were unable to obtain or use a human conjunctival SCC line for our injection. However, as noted previously, our SCC line retains many similarities to conjunctival cells and thus remains one of the best options available. Third, the PEARL imaging system we used is designed for small animals and thus limits clinical translatability. However, indocyanine green imaging systems are readily available in ophthalmology operating rooms, and further studies are needed to identify if such systems can be used to image panitumumab-IRDye800CW in an ophthalmic setting. Fourth, there are inherent fluorescent imaging limitations including limited penetration depth, as well as autofluorescence. Further studies are needed to assess the impact these limitations have in ocular tissue.

## Conclusion

To the best of our knowledge, this is the first murine model for conjunctival SCC, as well as the first use of panitumumab-IRDye800CW in ophthalmology. Overall, our subconjunctival injection technique was able to create a tumor model that invades the basement membrane and mimics invasive conjunctival SCC. We also found that panitumumab-IRDye800CW can be used in the ocular and orbital space with a robust TBR, indicating that it has potential for ophthalmic use.
